# Editorial: Precision information identification and integrated control: pest identification, crop health monitoring, and field management

**DOI:** 10.3389/fpls.2025.1759708

**Published:** 2026-01-08

**Authors:** Hui Li, Matthias Schumacher, Gerassimos G. Peteinatos, Demosthenis Chachalis, Wei Ma, Xiongkui He, Pei Wang

**Affiliations:** 1 College of Engineering and Technology, Key Laboratory of Agricultural Equipment for Hilly and Mountain Areas, Southwest University, Chongqing, China; 2 Department of Weed Science, Institute of Phytomedicine, University of Hohenheim, Stuttgart, Germany; 3 Soil & Water Resources Institute, Hellenic Agricultural Organisation—DIMITRA, Athens, Greece; 4 Laboratory of Weed Science, Benaki Phytopathological Institute, Kifisia, Greece; 5 Institute of Urban Agriculture, Chinese Academy of Agricultural Sciences, Chengdu, China; 6 College of Science, China Agricultural University, Beijing, China

**Keywords:** precision agriculture, information processing, integrated control, pest management, crop monitoring

In terms of intelligent monitoring and precise control of pests and diseases, related research has shifted from traditional visual inspection and single image recognition to a comprehensive technical approach that combines multi-source perception, deep learning and intelligent equipment. Focusing on the chain of “early monitoring - precise identification – quality and yield assessment - variable operation control”, literatures have respectively carried out systematic explorations in aspects such as acoustic and hyperspectral perception, three-dimensional phenotype acquisition, design of pest and disease identification models, and integration of variable spraying and intelligent operation systems, providing important support for the construction of a precise pest and disease control technology system.

In the field of multi-source perception and early monitoring, an acoustic-visual representation method based on cross-modal adaptation was proposed. By filtering, down sampling the insect acoustic signals and constructing patch-level log-scale mel spectrograms (PLMS), the acoustic signals are visualized and then connected to the pre-trained YOLOv11 (Wu et al.). Zhang et al. utilized hyperspectral and high-resolution RGB images from unmanned aerial vehicles, combined with ground investigations, to conduct index screening and severity classification for walnut leaf blight in southern Xinjiang. Random forest was used to achieve fine classification with an overall accuracy of 86% and a kappa of 0.825. Li et al. combined hyperspectral imaging with the 3D-2D hybrid convolutional network 3D-2D-LCNET for the first time. By selecting 15 to 30 feature wavelengths through CARS and SPA, they maintained a recognition accuracy rate of over 96% despite significant dimensionality reduction, which was significantly superior to traditional machine learning and single-modal networks. It reflects the advantages of “spectral selection + structural mixing”.

In terms of phenotypic and growth status monitoring, Shi et al. used unmanned aerial vehicles equipped with LiDAR to obtain three-dimensional point clouds in soybean fields, separated crops from complex backgrounds, combined watershed and k-means to achieve individual plant segmentation, and estimated LAI with machine learning. Cheng et al. proposed the RGB-D multimodal multi-task learning framework, which simultaneously predicted the weight, size uniformity and quantity of post-harvest strawberries in a single network. Ru et al. (2025) constructed the first image dataset of tea garden picking behaviors, precisely labeled five types of operation behaviors and two types of tools, and verified detectability on models such as YOLOv5s, providing a data basis for incorporating the “human-machine-operation behavior model” into intelligent tea garden management (Han et al.). Qi et al. (2025) used a super-depth of field microscope and a field investigation system to characterize the feeding behavior, time rhythm and leaf position preference of the tea bug. They found that the damage rate of large-leaf tea trees with high shade was significantly higher, providing a quantitative basis for ecological regulation and the breeding of resistant varieties from the perspective of pest-host-microenvironment interaction.

Multiple efforts have been made in the field of intelligent disease identification and lightweight models. Rana et al. introduced spatial and channel attention mechanisms into CNN to guide the network to focus on the leaf lesion areas of rice, achieving high-precision recognition of diseases such as brown spot disease, leaf blast disease, and rice leaf roller, while also meeting the real-time reasoning requirements of edge devices. Cai et al. proposed an IMNM model integrating ResNet improved structure, DCN and PPN, which achieved an accuracy rate of 98.55% in the identification of five types of pepper leaf diseases and 99.81% in cross-species tests of apples, wheat, rice, etc. Xu et al. introduced deformable convolution and multiple attention mechanisms in MobileNetV3_small to construct a lightweight DSA-Net, achieving a recognition accuracy of 99.12% on a dataset of 7,915 pea leaves with only 1.48M of parameters. Xu et al. proposed LiSA-MobileNetV2 by reconstructing the inverted residual module, introducing Swish activation and SE attention, which increased the recognition accuracy of 10 types of rice diseases to 95.68%, while reducing the parameters and computational load by approximately 75% and 48% respectively.

For complex meteorological and cross-crop scenarios, Qin et al. proposed the SIS-YOLOv8 model. Through the superimposed pruning and compression of Fusions Inception Conv, C2f-SIS and SPPF-IS structures, it was applied in the identification of early and late blight in potatoes and tomatoes. Raj et al. embedded CBAM attention in YOLOv8s and adopted dynamic task-aligned detection heads to construct the YOLO-ODD model, achieving real-time detection of four types of leaf diseases in Onions: Anthracnose, stem blight, purple spot disease and leaf roll disease. The accuracy is superior to that of YOLOv5 and basic YOLOv8. The parameters are only 11.1M and can be deployed on mobile devices. Wang et al. proposed the lightweight ELM-YOLOv8n, integrating the Fasternet Block with the EMA attention and detail enhancement detection head into the network. The number of parameters and computational load were reduced by approximately 44.8% and 39.5% respectively. Fu et al. designed a lightweight MHDI-DETR based on RT-DETR, introducing the MobileNetV4 backbone, lightSFPN and Focaler-GIoU loss function. In terms of maize disease identification, Wang et al. proposed the Maize-Rust model, introduced SimAM attention and BiFPN multi-scale feature fusion, and adopted DWConv lightweight convolution, achieving an accuracy rate of 94.6% and an average precision of 91.6% on the self-built dataset.

In the field of pest target detection and fine-grained identification, research has gradually shifted from “being able to detect” to “seeing carefully and distinguishing clearly”. Chen et al. proposed a key-fg DETR framework based on Transformer, integrating FGSP, MaskMLP, denoising module and DropKey strategy, which significantly improves the separability of the foreground region of camouflaging pests. Wang et al. constructed the Swin-Aarnet model based on the Swin Transformer, fusing local details and global context through the depthwise separable residual attention block and the global spatial attention module. Qian et al. proposed a fine-grained classification network that integrates multi-scale features and a hybrid attention mechanism. By using a parallel feature fusion module (FFM) and a hybrid attention module (MAM), it takes into account both local features and long-term dependencies. Its overall performance on datasets such as IP102, D0, and Li is superior to existing methods.

In scenarios with dense small targets and occlusion, Liang et al. proposed an improved YOLOv8 lightweight CTDA model for greenhouse cherry tomato detection. By using the LAWDarknet53 down sampling backbone, soft pool multi-scale pooling, and attention-driven dynamic detection head, the detection capabilities of small targets and overlapping fruits were enhanced. Under the conditions of only 6.7M parameters and 154.1FPS, mAP@0.5 is 95.3% and mAP@0.5:0.95 is 76.5%, which is suitable to be used as the visual front end of picking robots. Fotouhi et al. combined hierarchical transfer learning (HTL) with SAHI slice super inference to detect small insects on yellow plate traps. By pre-training YOLOv8 on large-scale insect datasets and adopting an image slicing strategy, the performance of small target recognition was significantly improved.

In terms of quality/yield estimation and variable operation control, some studies further transform the identification and perception results into executable management decisions. Ma et al. proposed the YOLOv11-GSF model for the density of small targets and light fluctuations in the maturity detection of greenhouse strawberries, introducing Ghost convolution, C3K2-SG module and F-PIoUv2 loss. A mAP of 97.8%, an accuracy rate of 95.99%, and a recall rate of 93.62% were achieved on the high-rack strawberry dataset. Cheng et al. proposed a multi-scale feature enhancement network MFEN that integrates learnable density maps (LDM) for estimating the yield of red cluster peppers. It is proved that “density map + lightweight network” can be used for high-precision prediction of crop yield. The aforementioned strawberry RGB-D multi-task quality assessment and soybean LAI estimation work (Shi et al., 2024b) have expanded the modeling path of “from multi-source observations to yield/quality/growth indicators” from the perspectives of multi-task and three-dimensional structure.

In terms of variable spraying and intelligent operation control, Luo et al. designed a variable spraying system based on RealSense binocular vision and improved YOLOv8n to address the low utilization rate of constant spraying pesticides on kale. The recognition results were mapped to spray volume control signals through the duty cycle - flow model. Jiang et al. took leaf area volume density (FAVD) as the variable spray decision parameter. By using the excitation audio and BP neural network to estimate the FAVD and adjust the PWM duty cycle, a small orchard sprayer integrating FAVD detection, execution and automatic navigation was constructed. Compared with the non-target invariant spray, it significantly improved the uniformity of canopy deposition distribution, reduce ground and rear losses, and save water by 64.50%. Chen et al. established a prediction relationship for the droplet size of rotating disc atomization by using high-speed photography and dimensionless numbers Re and St, and verified the good fitting of the modified Rosin-Rammler distribution to the particle size distribution of large droplets, providing theoretical support for improving the uniformity and control effect of spray through working condition optimization. Wang et al. proposed the lightweight YOLOv5M-SBSD model. With the support of ShuffleNetV2, CARAFE, BiFPN and SimAM attention, the mAP for tea bud detection in natural tea garden environments was achieved at 93.1%, while reducing the model size and computational load by more than 80%. A deployable visual solution was provided for the intelligent tea bud picking robot.

